# Qualitative study of patients’ decision-making when accepting second-line treatment after failure of first-line chemotherapy

**DOI:** 10.1371/journal.pone.0197605

**Published:** 2018-05-25

**Authors:** Jean-Louis Pujol, Benoît Roch, Caroline Roth, Jean-Pierre Mérel

**Affiliations:** 1 Thoracic oncology Unit Hôpital Universitaire Arnaud de Villeneuve, Montpellier Cedex, France; 2 Epsylon: Interdisciplinary properties and property dimensions concurring to patients’ decision making about subsequent line of chemotherapy research unit dedicated to human sciences and health, EA 4556, Université Paul Valéry Rue du Pr. Henri Serre, Montpellier, France; Leiden University Medical Center, NETHERLANDS

## Abstract

**Objective:**

Treatment failures in advanced lung cancer are frequent events affecting patients during or after first-line chemotherapy. International guidelines recommend second-line chemotherapy. However, around one half of patients who experience disease progression enter a systemic second-line therapy. In the herein qualitative study, we investigated patients' thoughts and attitudes determining the decision to undergo a second-line chemotherapy.

**Methods:**

Thirty-three purposively selected patients who recently accepted second-line or palliative chemotherapy were invited to participate in this survey consisting of semi-structured in-depth interviews. Grounded theory was applied to investigate participants’ perceptions of the context that have surrounded their decision to undergo palliative chemotherapy.

**Results:**

For most patients, tumor burden and reduced quality of life in relation with lung cancer itself were major drivers of the decision-making process. There was a balance between two different attitudes: making a decision to undergo a new line of chemotherapy or starting a psychological process in order to accept end of life. Choosing between these two attitudes allowed the patient to keep the matter of palliative care at a distance. Even in case of low chance of success, many patients who worried about their life partner's future would accept a new chemotherapy line. Some patients experienced ambivalent thoughts regarding social network, particularly about their family as daily function impairment required an increased need for relative's support. The initial "Worrying about others" thoughts left place to in an increasing self-need of care as those provided by relatives; this phenomenon might increase patients' self- perception of being a burden for others. Confidence previously established with formal caregiver support was another major decision driver: some patients with sustained confidence in their medical staff may have privileged this formal support rather than family support when the latter was perceived as weak, insufficient or intrusive.

**Conclusion:**

This study identified three domains involved into a complex interplay for lung cancer patients’ decision regarding second-line palliative chemotherapy: (i) perception of the definitive loss of health, (ii) interactions between idiosyncrasy (hope, disease burden) and environment (healthcare and social network support), and (iii) patient's subjective evaluation of chemotherapy benefit–risk.

## Introduction

Lung cancer is the leading cause of cancer mortality among women and men in most developed countries [1]. Most of the patients present with metastatic disease at time of diagnosis [2] and 80% of them suffer from high symptomatic burden [3]. Despite progresses in new strategies (immunotherapy, targeted therapy) a clear majority of patients requires chemotherapy as front-line treatment with palliation (*i*.*e*. symptoms alleviation and delaying the occurrence or progression) as a primary end-point [4].

For both small cell lung cancer (SCLC) and non-small cell lung cancer (NSCLC), biological resistances are frequently acquired by disease, during or after first-line chemotherapy. Whatever the pathological group of lung cancer, international guidelines recommend second-line chemotherapy [5,6]. However, only about one half of patients who experience disease progression enter a second-line systemic therapy [7].

Different reasons can explain why not proposing a patient a second-line therapy. Among which somatic conditions such as impairment of performance status, impaired end-organ functions or terminally-ill status, are obvious contra-indications to second-line treatment [7]. However, once having considered these somatic criteria for non-eligibility to second-line therapy, many questions remain about the patient’s decision making [8]. Active participation of the patient into the decision is strongly supported by the literature [9,10] and recommended by guidelines dealing with patient-caregiver conferences [11]. Nevertheless, this active participation is sub-optimal in many cases [12]. In a systematic review of patient’s preferences, it has been suggested that NSCLC patients agree to receive systemic therapy with low prognostic impact, but their decision depends on various parameters such as expected tolerance profile of the forthcoming chemotherapy [12].

In the herein qualitative study, we investigated patients' thoughts and attitudes that determine the decision to undergo second-line chemotherapy. Patients who underwent chemotherapy were interviewed using a semi-structured questionnaire in order to determine the features and cognitions occurring during second-line therapy with particular attention to patient-caregiver relationship, patient’s cognitions, hope, coping and interaction with the formal health care and informal social network.

## Methods

This qualitative interview study is part of a larger project that examines chemotherapy in the palliative phase of lung cancer, evaluates patients’ decision-making and compares them with views and attitudes of their relatives, caregivers and oncologists.

In the herein cross-sectional survey study, participants with lung cancer underwent an in-depth face-to-face interview to express their expectations and reluctances about the second-line program after failure of first-line chemotherapy.

Interviews were conducted by a trained-female psychologist (CR, master’s degree). She was employed halftime for the purpose of this study. Before this study, she had undergone a one-year training program in a cancer center (Institut du Cancer de Montpellier, Montpellier France).

The interviewer met the participants the day before the interview to offer them to participate in the study and to obtain their informed consent. Participants were frankly informed that participation in the study was their own choice and that refusal to participate would not modify medical and psychosocial care. To rule out any misunderstanding about the research goal, psychological support was provided by another psychologist who was not involved in this study.

### Study design

Grounded theory was applied to gather concepts and themes emerging from data [13,14]. Consolidated criteria for reporting qualitative research (COREQ) were applied [15].

#### Sampling

Participants from both genders and a wide range of age and socioeconomic status were sampled. The research was offered to consecutive participants admitted to receive chemotherapy (second-line or third-line / palliative intent) for a lung cancer in the thoracic unit of the Montpellier academic hospital (Arnaud de Villeneuve Hospital, Montpellier).Eligibility criteria consisted of being aged 18 years or older, receiving conventional (not investigational) chemotherapeutic drugs for treating either a small cell or a non-small cell lung cancer according to the latest American Society of Clinical Oncology guidelines [16], being fluent in French and having signed the informed consent form.

Patients were approached on day one of an ongoing second- or third-line chemotherapy program while they underwent the second cycle of the planned chemotherapy. Interviews were performed face-to-face, in one setting, in a quiet room and at a time when they did not undergoinvasive care or investigations.

Preplanned accrual population during the study was 33 patients. During the accrual period, 40 patients were contacted; among them 7 patients disagreed to participate mainly by fears that the interview would increase their preexisting anxiety.

Participants’ characteristics are summarized in Table 1. Thirty-three patients participated in the study; sixteen were interviewed during the second-line of chemotherapy; seventeen participated in the subsequent line / palliative chemotherapy prescribed to circumvent failure of a previous line of chemotherapy (progression of the disease occurring after transient improvement).

**Table 1:** Patients' demographic and disease characteristics.

Patients' characteristics	N (%)
N	33
Age (mean in yr)	61.8 +/- 7.9
Female n (%)	11 (33)
Significant others	19 (71.4)
Partner, parent or sibling	15
Others	4
Histology	
Small cell lung cancer	6 (18)
Non small cell lung cancer	27 (82)
Progression free interval since previous line	
More than three months	20 (61)
Less than three months	13 (39)

#### Study approval

The research protocol and the semi-structured interview were submitted prior to study activation to the *Comité Consultatif sur le Traitement de l’Information en matière de Recherche dans le domaine de la Santé* and both received approval (CCTIRS 15.472). The study was approved by the ethics committee of *Montpellier-Méditerrannée*.

#### Interviews

The semi-structured interview consisted of eleven questions (appendix 1). A first version was tested during the analysis of the five first interviews. No rewording was needed and the same questionnaire was used throughout the study. Probes were prepared by the interviewers and used in order to promote open answers. Semi-structured interview goals were as follows (i) detecting pre-existing conditions that construct patients’ knowledge about the palliative endpoint of the second-line therapy; (ii) restarting from that point, determining personal changes (*e*.*g*.coping, hope) and environment changes that buildanevaluation of risks and benefits induced by a new chemotherapy and (iii) to explore the decision-making process about accepting a subsequent line of chemotherapy.

Interviews were audio-recorded with participants' permission, and conducted in only one session. They lasted from 40 to 90 minutes. Audio-recordings were then turned into verbatim transcripts.

### Analyses and findings

Transcripts were reviewed by a team of research coders. Participants' interviews were evaluated line-by-line using a text driven inductive approach to allow themes to emerge. Emerging patterns and themes were analysed with an initial open coding method. They were then clustered into axial coding categories. Afterwards, categories were refined into more abstract themes. Reliability of coding was secured through consensus agreement of the coders. We used a data collection process based on initial concepts derived from first interviews. Data collection and analysis process were conducted in parallel. This first step of analysis generated concepts. The initial concepts leaded to new questions emergences that drove the forthcoming data collection. As defined by theoretical sampling, during this eighteen-month research, we continued this circular process until we considered that the point of saturation was reached, i.e. no new concept emerged and each concept was developed in terms of their properties and dimensions. NVivo Version 10 (QSR International Pty Ltd) software was used to code and explore the relationships between concepts.

## Results

### Inductive reasoning

#### “Patient- medical staff relationship” and “experience about first-line treatment” as contextual conditions

**Fig 1:**
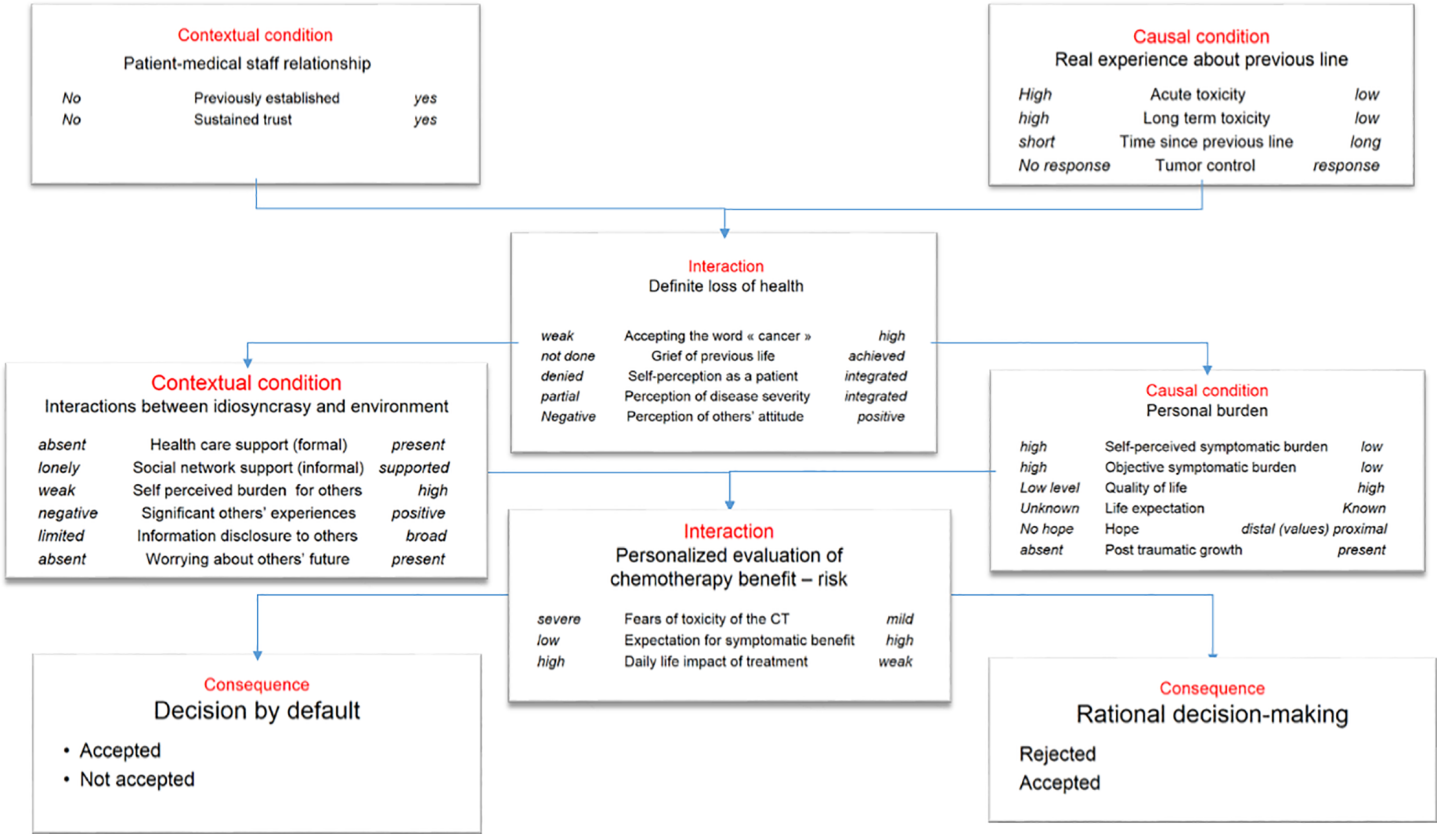
Categories, properties and property dimensions concurring to patients’ decision making about subsequent line of chemotherapy. Categories and dimensions have been arranged in a final tree diagram structure in the analytical framework. Interpretive concepts and propositions emerging from clustering categories described three themes: (i) perception of the definitive loss of health, (ii) interactions between idiosyncrasy (hope, disease burden) and environment (healthcare and social network support), and (iii) patient's subjective evaluation of chemotherapy benefit–risk.

The herein investigated phenomenon was the interaction between *patient—medical staff relationship* and *experience about first-line treatment* (as both preexisting conditions) in patients who had accepted to undergo a second-line treatment after failure of first-line chemotherapy (Fig 1).

The main category that emerged in the open coding part of the study was *the perceptive definite loss of health*. Consequently, this category has been labeled as a strategic action / interaction category and has been used as the axis for the axial coding part of the study.

#### “Personal burden and hope” as causal condition

We systematically sought the condition categories and the explanatory pathway towards the aforementioned action / interaction category (*participative definite loss of health*). There was a set of conditions that defined the context of action / interaction. Some contextual conditions belong to *personal burden and hope* (*e*.*g*. burden of the disease, coping); other contextual conditions belong to the *environment* (*e*.*g*.health care support and social network support, but also significant others’ experience). Other condition categories are intervening conditions (*e*.*g*.*life expectation*, *post traumatic growth following the first line*). Each of the conditions categories were developed in their properties varying into a broad range of dimensions. The *knowledge of the disease*, which greatly contributed to the *perceptive definite loss of heath*, has been detected as an important causal condition.

We then investigated explanatory ways of action / interaction towards consequences, namely, acceptance of second-line therapy. The condition categories were interwoven in a complex and singular way towards a secondary important action / interaction, namely *chemotherapy risk-benefit balance*. Although this study only accrued patients who have accepted the chemotherapy, it appeared that acceptance varied in degree from a *rational decision making* to *a decision by default*.

### Emerging domains

As a first step, open coding identified eight categories shown in Fig 1. There were validated by axial coding and we defined three domains that provided insights into the participants’ views and attitudes towards decision making: (i) perception of the definitive loss of health, (ii) interactions between idiosyncrasy (hope, disease burden) and environment (healthcare and social network support), and (iii) patient's subjective evaluation of chemotherapy benefit–risk.

### Perception of definitive loss of health

#### Balance between acceptance of being at the end of life and the agreement to receive chemotherapy

There is a balance between two different attitudes: making a decision to undergo a new line of chemotherapy or starting a psychological process in order to accept end of life. Some patients seemed to discuss freely about end of life and euthanasia, but this discussion was facilitated by the existence of a second option: as long as continuing active anticancer therapy remained possible, these patients were able to speak about their end of life. The choice between going on chemotherapy and refusing it, allowed the patient to keep the matter of palliative care at a reasonable distance. When patientsmade a decision not to go through a new chemotherapy line they surely knew that the only remaining possibility is palliative care (*e*.*g*.pain alleviation, nutritional support). Consequently, the decision to reject chemotherapy was a surrogate to the acceptation of end of life.

“There, from the day he got his cancer… He never felt lively again. And that’s what scares me a bit today. Before I even thought about it… for me, cancer, as I knew nobody except Alain, was for me a disease like any other.”

#### Disease changes towards metastatic status

Facing lung cancer metastases outside the thorax, particularly when the local cancer was paradoxically controlled, was a difficult task since the modification of disease status was concomitant with changes into its representation. For instance, patients with a relapse consisting of brain metastases and no relapse to the chest were almost facing a new cancer.

“Hmm… And yeah, last time, I’m going to see the professor and I think he’s going to tell me: “Six month remission”… and bad luck! He said: “Boom! It’s gone to the brain and liver”. And I was so…disappointed that I said: “I’m stopping everything! I don’t want anymore! I’m fed up! I fought until today…” I didn’t want anything more, I had lost faith.”

#### Different meanings of surgery and chemotherapy

Paradoxically, chemotherapy induced a more potent biographic rupture than surgery. Surgery was usually followed by a short recovery period and a return to normal life. Chemotherapy had frequently an unplanned end or could resume after a rest period so that its impact on the patient's life story was more pronounced. The first might appear as suspension of life continuity, the latter was mainly a biographic rupture with no return to the previous life.

“The first thing I asked the surgeon was: “Could I go to India in two month?” The first question was: “Could I leave? He said yes”. I would have felt bad if I could no longer travel. At times I think about that.”

Grief of previous life is a fourth property of this domain (see appendix 2).

### Interactions between idiosyncrasy and environment

#### Worrying about other’s future and self-perceived burden to others

Even in case of low chance of success, a patient who worried about his life partner's future would accept a new chemotherapy line. When empathy with his significant others was involved, a positive decision making was the most probable response.

“There you have it. But well, I live a little in fear… more than him. But that’s normal. I guess that for the one that’s left behind, it’s something else…the fear of losing him, the fear of life without him…”

With the end of life as unique perspective, a patient might have ambivalent thoughts about social network especially about the family. On one hand, his anxiety may increase in regards to family's future, and, on the other hand, with daily functional impairment, the need for support might increase. The latter aspect of the patient-caregiver relationship became more important over time with quality of life impairment. As a matter of fact, "worrying about others" left place to a self-perceived need for caregiving from relatives.

“I have to… As long as I’m well, self-sufficient, that I don’t have to ask anymore, because I don’t want to ask anybody, it will be alright… But when I’ll be relapsing, maybe even during the treatment, there’s going to be…Uh, I know, there’s going to be a time when I’m going to say: “Stop, we stop there and it’s over!” Here… And when I say it’s over, it will be over. There’s no one who can change my mind.”

#### Ambivalent relationship with informal (social) support and significant others

There was no gold-standard attitude from the social support that surrounds patients suffering from lung cancer. An attitude could be considered as intrusive at a given time, whereas in other circumstances, the same attitude could be a patient's absolute request.

Diagnostic disclosure to others lead to a constraint for the patient: once he had adopted an attitude of diagnostic disclosure, he would be facing new events over time, such as treatment failure or metastatic progression of the disease, which might require new disclosures. Even then, patients tried to maintain the same disclosure attitude. This new state required new coping strategies and patients were enforced to face worsening state and poor prognosis. Consequently, some patients with sustained confidence in their medical staff, might have privileged the formal support rather than family and informal support when the latter was perceived as weak, insufficient or intrusive.

“Yes and then Professor P. said to me: “But why doesn’t your mother come?” “No”, I said, “no, I don’t want her to, I want to be alone”. But with this medical team I don’t need…they really listen, they’re gentle, they’re… I have, I have something, well, I know that… I say “it hurts there”, they’ll tell a nurse and a nurse will pass by.”

Conversely, intrusion (morbid thoughts) was favored by loneliness. Many patients experienced distress and anxiety when being alone and required a support by some social network and sometimes even partner's silent presence in order to avoid these intrusive cognitions.

“Now there are always my anxieties. The hardest moments are the moments when I’m alone at home for a longer time as usual. While someone is there I don’t think about it. When I’m alone for a long time, even I don’t want it, I have sneaky thoughts.”

Other properties belonging to this domain were *decision* delegation to others and patient’s self-image perception (see appendix 2)

### Personalized evaluation of chemotherapy benefit–risk

#### Patients' definition of framework for acceptance

Some patients accepted chemotherapy as a general concept, and others made different processes regarding chemotherapy method and treatment goals. For the latter setting, the decision making might have been conditioned by distal endpoints: primary endpoint being the positive effect of treatment (such as alleviate symptoms, improve quality of life and expectation for prolonging life); the second endpoint was that no inactive chemotherapy must be maintained. However, the first issue regarding drugs and chemotherapy schedules clearly belonged to medical staff and its acceptance by patients was sustained by confidence.

“When they tell you you have a disease, you know that means chemotherapy. They say “cancer” you think “chemotherapy”! When they tell you you have a cancer, you know you will have chemotherapy. You’ve got to trust them. The decision is not yours. After that, choose chemotherapy? Yes, I chose it, but I said that I didn’t want overtreatment. I would choose to stop it or something… There has to be trust: whenI arrived in the service, in front of doctor R. I said: “Here is where I want to be treated.” They are the ones who can cure you (the doctors).”

During decision making patients needed information. But at a given time there was a need to decide whether or not the therapeutic proposal might be accepted. There was a kind of fatigue that limited the consciousness of the decision. Therefore, the acceptance of the proposal was more a health professional induced choice than a shared decision.

“I actually do not doubt, should I say that? … I trust the doctor completely. If he decides that he should change the treatment for such or such reason…well… we discuss it. Sometimes I give up. But if that’s what it takes, then you have to. I know it’s for the best.”

Two attitudes have been observed: The first one was a slow process along a formal pathway from initial proposal of chemotherapy for acceptance. This pathway needed knowledge acquisition. During this process, participants' cognition balanced between 2 thoughts: “I should accept” in order to go forward, and “I need searching” in an attempt to consent to chemotherapy protocol in full knowledge of the facts. This back and forth reflexions allowed participants to manage personal views with medical discourse regarding treatment by slowing down the acceptance process. As a final result, interviews demonstrated that knowledge participated in the trusted process that helped maintain a positive perception of the future. Therefore, the process might hesitate during an undefined time between the two categories: *rational decision* or *decision by default*.

#### Expectation of symptomatic benefit

Most of the patients followed a rational process towards decision making about acceptance of chemotherapy so that the decision depended on objective risk-benefit ratio evaluation, step-by-step. For instance, the treatment was continued as long as the symptoms were alleviated. This rational process enhanced the self-perception for patient of being involved as a partner into a shared decision.

“Finally we have to set limits. In addition we have the right at least to refuse the treatment but we do not have the right to say stop afterwards. So we should have a choice for everything.”

Several parameters (variables) entered into account when a patient rationally decided to accept treatment: the first one was a risk-benefit evaluation considering both symptomatic alleviation and toxicity profile of the new therapy. The second variable consisted of benefit expectation in terms of duration. As a matter of fact, the longer the expected benefit was, the more the patient had confidence in the new treatment.

This phenomenon was observed even in case of limited expectation (about one year) because the expected duration only came from a probability calculation so that patients hoped for entering the subgroup of individuals who benefit from the longest therapeutic activity. In addition, going from one line to the next also depended on the positive or negative experiences about the previous one. A success of the previous line, even if it were transient, made it possible to consider the forthcoming chemotherapy as a new opportunity to sustain hope.

#### Expending time since the first-line

There was a discrepancy between patient's perception of therapeutic change and medical aim sustaining this new treatment proposal. On one hand, when the first-linetreatment failed before going to the end of a complete program, (*i*.*e*. a disease progression occurring early in the treatment program), the new treatment line was considered as a salvage therapy and was understood by the patient as a mandatory modification of the same therapy (*i*.*e*.as if it were the same line of treatment): in this case patient's view occulted the failure of the previous chemotherapy line. On the other hand, when a significant time had elapsed between the first and the second-line treatment a complete reasoning was needed before the patient made a decision. Thus, a gap between the first-line and subsequent therapy was needed for a rational decision-making, whereas the lack of splitting in the rush of the therapy limited the expression of the patient's desire.

“Because I had pushed everything out of my head, everything I had suffered, all the bad things I had suffered, I had pushed everything away: for me the disease was gone,I had gone on to something else. I was doing sports to strengthen my muscles.”

#### Treatment induced adverse events as surrogate for “cancer”

Some patients had cognitions of intrusion of being divided: a part of them named as "tumor" and another part that continued living as a self-rid of the disease. Adverse experiences induced by treatments were memos signifying to patients, their “forgotten part”, namely themselves as cancer patients. Chemotherapy induced adverse effects were surrogates of the disease that were either asymptomatic or at least did not impair quality of life.

“And I confess, it was worse for me when I was told that I would lose everything, my hair, my beard, and all that, than when I found out I had a cancer. I give you my word that’s the truth.”

“Often I feel like I have these tumors that are going in the bad direction, and there is me who continues to be active. Except during the very hard chemotherapies where you have to remain in bed. It’s true that I’ve had some very long chemotherapies. I couldn’t take anymore.”

The negative experience of first-line-induced toxicity did not automatically lead to refusal of or reluctance about the second-line therapy. The capability of some patients to overcome fears about toxicity might be linked to some factors such as: coping with the new life conditions, post traumatic growth and hope in symptomatic benefit (*e*.*g*. prolonged life expectancy).

“What I want is to be well treated. I don’t give a damn about my hair…”

#### Fears of chemotherapy toxicity were balanced by the expected symptomatic benefit

In the decision-making process, the expected toxicity induced fears. However, as stated by secular knowledge about chemotherapy, toxicity was directly linked with efficacy, (*i*.*e*. an intensive treatment would give better anticancer effects than another one with lower adverse effects). Actually, the relationship between toxicity and activity is a medical reality. Generally speaking, chemotherapies given at curative intent are frequently intensive with survival as a primary endpoint, whereas palliative chemotherapies mainly attempt at symptom alleviation and at a quality of life improvement and consist of drugs with better safety profile. This information was well-known by patients who emphasized this link into an equation: no toxicity—no effect.

“… Because I am against drugs. Because I rejected them, and so, I say to myself they destroy so much alongside. Maybe they heal something, so to speak, but they destroy so much else.”

Sequence of chemotherapy in a multimodality treatment was a sixth property of this domain (appendix 2)

## Discussion

In the herein qualitative study, we identified three domains involved into a complex interplay for lung cancer patients’ decision regarding second-line palliative chemotherapy: (i) perception of definitive loss of health, (ii) interactions between idiosyncrasy (hope, disease burden), environment (health-care and social network support), and (iii) patient's subjective evaluation of chemotherapy benefit–risk.

Reduction of medical care aggressiveness for cancer patients in palliative situation is an important challenge of medical oncology [17]. Quality of medical care delivered to cancer patients near the end of life is evaluated by several criteria. Lack of use of hospice and palliative unit resources is a main one [18]. Use of chemotherapy in the 3 last months of life or alternatively start of a new systemic line of treatment during the last month of life is another main criterion [19] and mainly affect ethnic minorities [20]. Attention of medical oncologists at the overuse of chemotherapy as a key determinant of poor quality of near the end life has been highlighted by the Agency for Healthcare Research and Quality and other health agencies or scientific societies. Overly aggressive care could be avoided by taking into account patient's attitudes towards chemotherapy, and expectations from a new line of treatment and physician—patient shared decision. The herein study might help both physicians, patients and their family to better understand each other.

Patient-doctor relationship, as it has been previously established during first-line chemotherapy, is a cornerstone of the decision; the patients’ experience about previous treatment is a second important driver. Both themes are important preexisting conditions leading to the *perception of the definitive loss of health*. Strategies to cope with this reality are strongly interacting with the patient environment, mainly social support, but also health care formal support. These current conditions actively construct an *individual chemotherapy risk-benefit evaluation*. This process needs time and a particular attention from professional caregivers.

It is well known that the distress caused by cancer has a profound effect on the daily lives of patients and their families [8]. Receiving care for cancer can lead for some patients to the sense of having become “*a burden to others*”. This *self-perceived burden* can influence exchanges within care giving relationships and also affect how patients adapt to the functional and psychosocial changes [21]. Our study suggests that *self-perceived burden for other* is an important condition in the decision-making to receive palliative treatment. The other major driver of the decision consists of *symptomatic burden of the disease itself*. For this latter condition, the case for lung cancer does not differ from other advanced malignancies. For instance, in women affected from breast cancer, the importance of these factors in psychological adjustment has been demonstrated [22]. According to a multiple regression analysis, factors associated with the need for psychological support mainly included emotional distress due to the need to adapt to rapid worsening of the breast cancer patients' condition [22]. Helping the patients' coping strategies is an important goal inasmuch as patients cope significantly better with primary systemic treatment by strengthening their coping strategies rather than resigning themselves and/or refusing social support [23].

### Previous experiences challenge the evaluation of second-line therapy

The expected benefit-risk ratio of the new therapy is not the only parameter to be taken into account. The experiences of previous line and even more the expected increase or decrease in toxicity of new therapy are other important variables. We define three different clinical settings: (i) The toxicity and the impact on daily and social life of first and subsequent lines do not differ significantly: this situation is more a restart of the known therapy rather than a new line. (ii) The toxicity and impact on daily life greatly differ: in this case the thoughts and attitudes completely change depending on the sequence: going from the highly toxic chemotherapy to an easier therapy, promotes the decision-making. On the contrary, leaving an easily accepted treatment (such as daily oral tyrosine kinase inhibitor) for a conventional chemotherapy (inducing hair loss, frequent returns in the therapy unit, venous punctures, etc.), is a difficult task for the patient; it requires more time in the decision process. (iii) The third clinical setting is maintenance chemotherapy consisting of reducing the number of drugs after an induction period. Accepting the meaning of maintenance is subjective and idiosyncratic: some patients perceive maintenance chemotherapy as a reduction in the treatment burden; others suffer from the lack of precise end of the chemotherapy program.

An extended time without symptom and without toxicity since the end of the previous line is not a guarantee that the patient will easily accept to return to treatment. On one hand, the disappointment about previous line treatment failure might overwhelm patients with negative feelings; on the other hand, the previous experience of a long remission of the disease might increase their trust and hope in the therapy efficiency.

### Coping with uncertainty

Some patients use rationalization in order to circumvent the general features of the disease. They hope that their own cases have a better profile in comparison with general agreement about lung cancer [24]. Several parameters (variables) enter into account when a patient rationally decides to accept treatment: the first one is a risk-benefit evaluation considering both symptomatic alleviation and toxicity profile of the new therapy. The second variable consists of benefit expectation in terms of duration. As a matter of fact, the longer the expected benefit is, the more the patient has confidence in the new treatment. This phenomenon is observed even in case of limited expectation (about one year) because the expected duration only comes from a probability calculation so that patients hope for entering the group of the longest therapeutic activity.

### The decision-making process cannot be totally rational

Interactions between idiosyncrasy and environment in making a decision are complex and vary from case to case. In order to simplify the herein qualitative study results, this could be written as an equation *ax+b = y* where *y* is the decision to accept or reject the second-line of treatment. Let *a* represent a personal burden and hope; let *b* represent environment that could take a negative or positive value, and may contribute to the decision (taking into account significant others' attitudes, support of the formal network, etc.). Interaction with environment is complex and a lack of environment is by itself a negative factor. Let *x* represent an unknown variable, which is not disclosed and probably not fully conscious for the patient. It could be compared to an intuition that the treatment could be beneficial or not. If *x = 0*, then decision only depends on *b* and that could explain that some patient accept or reject the second-line whatever the personal burden is.

### Study limitations and trustworthiness

There are limitations to this study. Firstly, the qualitative nature of the study does not allow considering the different concepts to be definite demonstrations. However, these concepts are hypothetical drivers regarding construction of decision. Secondly, only patients who accepted second-line chemotherapy have been interviewed so that the statements cannot be extrapolated to the whole population of patients to whom this palliative chemotherapy is proposed. Moreover, to interview patients who have refused chemotherapy would have been problematic inasmuch as patients attending the thoracic oncology unit do so to receive anti-cancer treatments. In a forthcoming phase of our project we plan to interview patients admitted for supportive care only, into the palliative department of our institution.During the study process, we did not harvest quotes that could counter the developing theory. The question regarding possible contra-examples of the main theory as developed here deserves further investigations. Finally, the limited number of patients purposively interviewed in this study does not allow multi-parametric analysis of the different ways of decision-making.

Evaluation in qualitative research is a critical point. No single definition exists regarding the quality of the theoretical results as the one produce in the herein study. Several definitions have been given to the concept of quality evaluation: rigor, trustworthiness, validity, reliability, credibility and truthfulness have been required in methodological publications and recommendations but no harmonized list of criteria has been proposed. Hitherto, the most accepted recommendation is the COREQ requirement. The herein study fulfilled 30 out of 32 criteria of the COREQ criteria (all except the two criteria related to focus group that we decided not to perform due to the risk of participants’ anxiety enhancement). As authors of the herein manuscript, it is difficult for us to make an unbiased evaluation of the rigor and trustworthiness of our work. Nevertheless, several quality criteria might be checked in the herein report: reasonable claims based on evident observations were exposed, the scope of the research was delineated, participants’ actual words and concepts were used each time it was adequate, selection of participants was presented, and connection with the current literature has been provided. Therefore, this study complies with most of the recently published criteria of rigor [25].

## Conclusion

Patients eligible for second-line chemotherapy for lung cancer perceive the meaning of such a palliative treatment as a definite loss of health. Therefore, they confront a balance between two different attitudes: making a decision to undergo a new line of chemotherapy or starting psychological processes in order to accept end of life. Important drivers in the decision-making process are the previously established trust with the professional caregivers (during the first-line chemotherapy) and attitudes towards significant others, mainly self-perceived burden for others and worrying for the partner's future. These views and attitudes should be known by caregiver staff as patients need time to share their decision.

## Appendix 1 Open questions are typed in italic and closed questions are typed in roman

1. How do you define your illness and condition?
2. Have you had any effects of previous chemotherapy?
3. What relationships do you have with the health care providers?
4. Do you feel that the decision about treatment is taken by others than you?
5. What effects did the disease have on your daily life?
6. Do you feel supported?
7. Do you perceive a difficulty in the fact that others help you? Do you speak about your illness?
8. How does your family react when you say that you are receiving chemotherapy?
9. Did you know someone in your family who received chemotherapy?
10. What are your hopes or fears regarding chemotherapy that you are about to receive?
11. Why do you accept or reject the chemotherapy that has just been proposed to you?

## Appendix 2: Additional results

### Grief of previous life belongs to perception of definitive loss of health domain

Patients determined what was acceptable and unacceptable as a daily life taking into account the symptomatic burden (pain, fatigue, loss of autonomy, etc.). They sometimes expressed the possibility of suicide as an escape from an unacceptable state. However, over time, what was considered as an unacceptable treatment became finally a possible way of life (by coping) and the border was moved further. In this context, the significant others' opinion might be more important than personal morbid ideations in refraining the action itself.

“So I even considered… I even foresaw… Finally I did not say much to my entourage, to put an end to it all, if I saw that it was… Then I considered two or three solutions… Foolish stuff you know, but, because I told them I never put up with being handicapped, suffering well… Suffer, the medics don’t let you suffer nowadays, but if I see that I am too much handicapped… to put end to it all, and to explain why. I thought about it, but more than just thought, I planned everything in detail. That means weapons, stuff for me… between us…”

### Delegating the decision to others belongs to interactions between idiosyncrasy and environment domain

There was a balance between giving up with heavy treatments (and accepting a shorter life expectancy) and continuing living with reduced quality of life due to chemotherapy. Some patients considered that the reasons for accepting the latter option mainly belonged to their relatives' expectations rather than to their own desire.

Most of the time, when a decision was taken by default, *i*.*e*. when a shortcut occurred in the patient decision-making process towards a passive decision, the patient's decision was compliant with the medical staff proposal. Rejection was usually constructed against the medical staff proposal and was a personal and active decision making.

“Because it’s a way for me to… uh, but it doesn’t necessarily work for everyone, but it’s a way for me to be involved in my treatment. I refuse to be a piece of flesh, facing a medical staff that knows everything. I’m not like that!”

### Distortion between patient’s self-image perception and others' attitude belongs to interactions between idiosyncrasy and environment tdomain

Some treatments given in order to palliate adverse effects of chemotherapy *i*.*e*. steroids, changed positively the patient's appearance. Then, what the patient considers as a difficulty (for instance facial edema) was supposed by others as a sign of better health.

“There are side effects … what disturbed me the most was the edema, which I saw in the mirror. They tell you « you look good » but you know deep down that this is not the truth.”

### Sequence of chemotherapy in a multimodality treatment belongs to personalized evaluation of chemotherapy benefit–risk domain

Attitudes towards chemotherapy in multimodality treatment differed according to the sequence of chemotherapy and surgery. When the surgery was the first step of the multimodal treatment, most of the patients considered that the radical treatment has been already done at the time when adjuvant chemotherapy was proposed and its significance recovered as a "preventive" goal. Conversely, when the surgery was planned after preoperative chemotherapy, patients seemed to experience more difficulties in considering surgical time as a radical treatment time.

“It’s traumatic like an accident, but at the same time when I started the chemotherapy I stopped feeling good, I no longer had a tumor… “Preventive chemotherapy” they said. I thought: “so it will be alright, it’s a preventive chemotherapy”…”
